# Cervical Epidural Hematoma after Chiropractic Spinal Manipulation Therapy in a Patient with an Undiagnosed Cervical Spinal Arteriovenous Malformation

**DOI:** 10.7759/cureus.307

**Published:** 2015-08-18

**Authors:** Meng Huang, Sean M Barber, Marc Moisi, Suzanne Powell, Andreana Rivera, Michael Zwillman, James Rose

**Affiliations:** 1 Department of Neurogurgery, Houston Methodist Neurological Institute; 2 Neurosurgery, Swedish Neuroscience Institute; 3 Department of Pathology and Laboratory Medicine, Houston Methodist Hospital; 4 Anesthesia and Critical Care, Houston Methodist Hospital; 5 Department of Neurosurgery, University Medical Center Brackenridge

**Keywords:** spinal epidural hematoma, chiropractic manipulation, spinal arteriovenous malformation

## Abstract

Spinal epidural hematoma (SEH) occurring after chiropractic spinal manipulation therapy (CSMT) is a rare clinical phenomenon. Our case is unique because the patient had an undiagnosed cervical spinal arteriovenous malformation (AVM) discovered on pathological analysis of the evacuated hematoma. Although the spinal manipulation likely contributed to the rupture of the AVM, there was no radiographic evidence of the use of excessive force, which was seen in another reported case. As such, patients with a known AVM who have not undergone surgical intervention should be cautioned against symptomatic treatment with CSMT, even if performed properly. Regardless of etiology, SEH is a surgical emergency and its favorable neurological recovery correlates inversely with time to surgical evacuation.

## Introduction

Spinal epidural hematoma (SEH) is a rare phenomenon. Although the etiology of spontaneous SEH is poorly understood, a close association with trauma, coagulopathy, and iatrogenic causes, such as spine surgery, epidural catheterization, and lumbar punctures has been observed with non-spontaneous SEH. Even more rare is chiropractic spinal manipulation therapy (CSMT)-induced SEH, and to the best of our knowledge, there are currently only 11 other published cases [1-11]. Our case is unique because the patient had an undiagnosed cervical spinal arteriovenous malformation (AVM) revealed on pathological specimens from the evacuated hematoma. 

## Case presentation

Informed patient consent was obtained from the patient described in this case presentation. No identifying patient information is contained in this paper.

**Figure 1:**
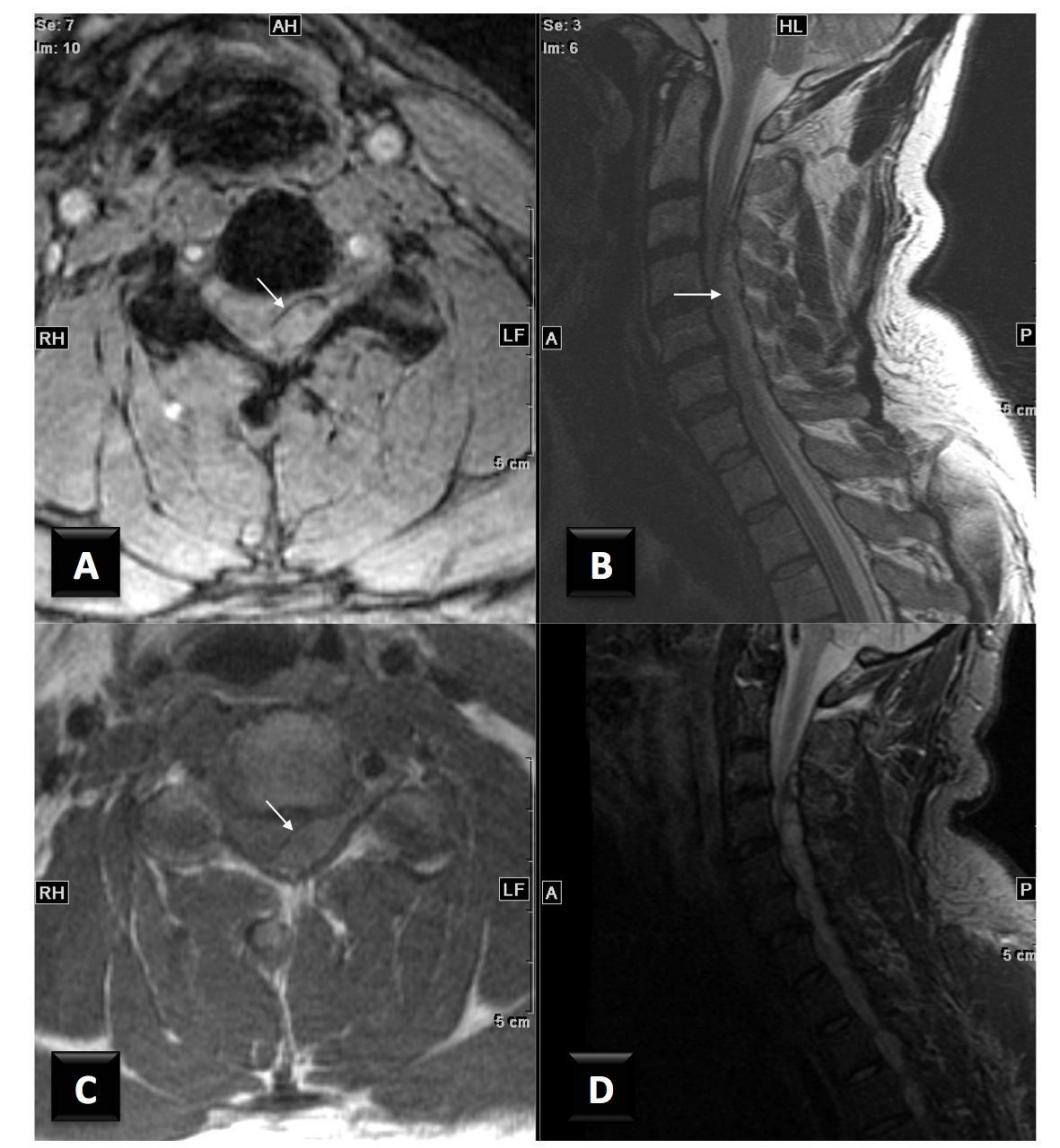
A) Axial T2 GRE image, B) Sagittal T2 FSE image, C) Axial T1 SE without contrast. The images show an extradural heterogenous collection (arrows) in the left posterior epidural space, which extends from C2 to C6, compressing the thecal sac and spinal cord. The mass demonstrates heterogenous T2 signal and isointense to slightly increased T1 signal centrally with rim of susceptibility representing blood products in an epidural hematoma most likely in the acute to early subacute stage.

**Figure 2:**
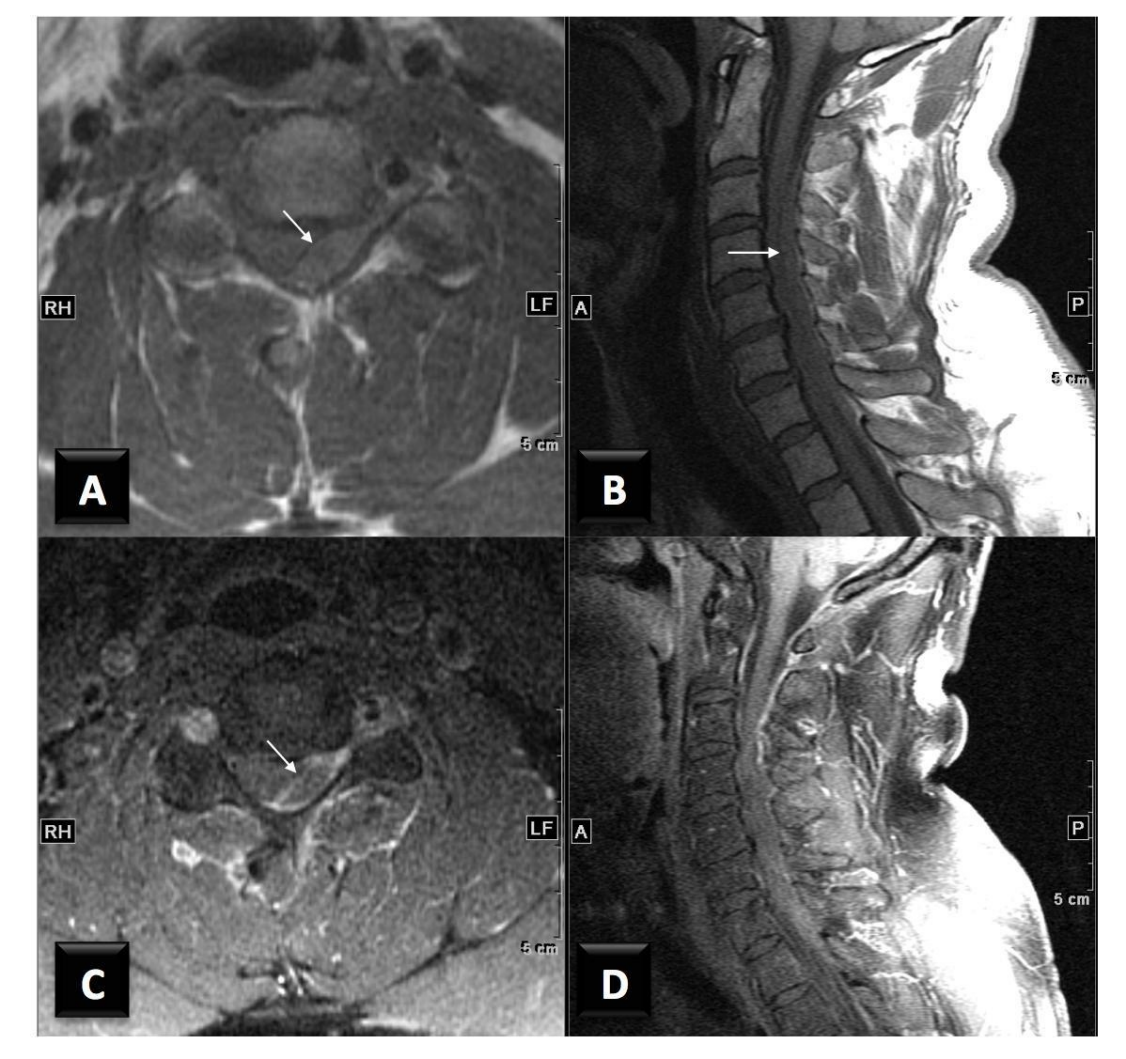
A and B) Axial and sagittal T1 SE images without contrast, C and D) Axial and sagittal T1 SE images with contrast and fat saturation. The images show the extradural heterogenous collection in the left posterior epidural space, which demonstrates isointense to slightly increased T1 signal with minimal peripheral enhancement (arrows), displacing the spinal cord laterally.

The patient is a 40-year-old male with no significant medical history who presented with a one-day history of severe neck pain and weakness of the left arm and leg. He underwent CSMT the day prior to admission, and the onset of pain occurred immediately following the CSMT session. He was not taking warfarin (Coumadin), aspirin, clopidogrel (Plavix), or any other anticoagulation agents. Admission INR was 1.0 and his platelet count was 320,000. The patient reported a slight decreased sensation weakness on his left side that began acutely approximately five hours prior to coming to the hospital. On physical examination, he was found to have marked weakness of the left upper extremity. His left triceps strength was 4/5, his grip, finger extension, wrist flexion, and extension were all 3/5. The motor strength of the left lower extremity was between 3 to 4 out of 5 throughout. The strength of both right upper and lower extremities was fully intact. An MRI of the spine identified a large posterior epidural hematoma extending from C2 to T2, displacing the cord anteriorly and laterally to the right (Figures 1-2).

**Figure 3:**
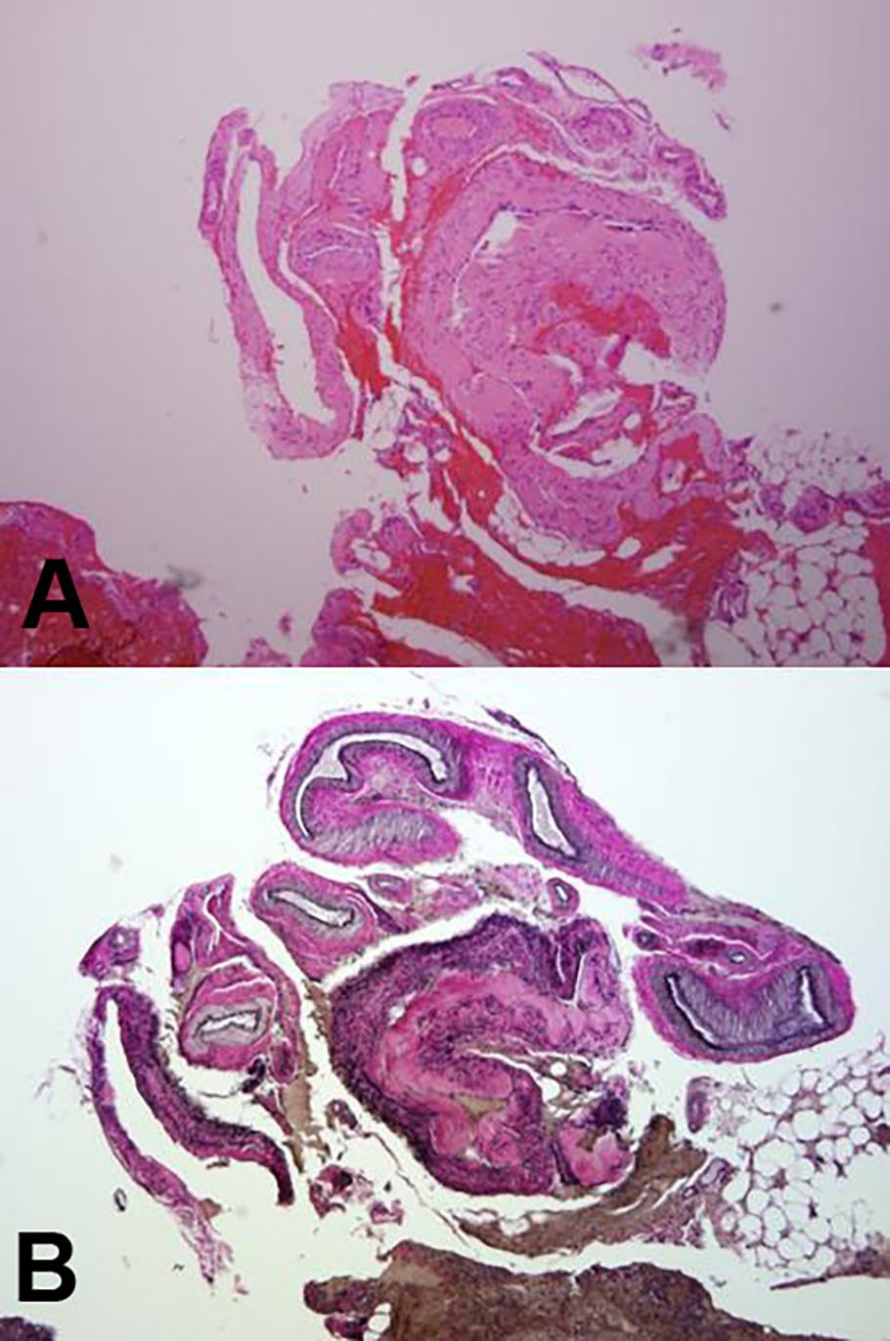
Pathologic specimens and staining pattern A) Hematoxylin and eosin stains of AVF displays a collection of variable caliber blood vessels, some with identifiable internal elastic laminae. B) Verhoeff - van Gieson (VVG) of AVM highlights the internal elastic laminae

A CT angiogram showed normal cervical vasculature. A formal spinal angiogram was not performed due to the development of a left hemiplegia necessitating surgical intervention. Approximately 18 hours after initial radiologic imaging, a left C3-T1 hemilaminectomy was performed with a solid hematoma evacuation. There was no obvious source of active bleeding found at the time of surgery. A specimen from the hematoma was sent for pathological evaluation. Pathologic examination of the hematoma specimen highlighted an arterial component utilizing Verhoeff-Von Giesen (VVG) staining for elastic fibers. The patient was found to have a spinal AVM (Figure 3a-3b). Postoperatively, his strength improved to 4/5 in his left upper extremity and 4+/5 is his left lower extremity. He was able to ambulate on his own by postoperative day 3 and was discharged the following day. A follow-up digital subtraction spinal angiogram two months postoperatively failed to identify any other vascular abnormalities. The patient had a minor cerebral thromboembolic event after the angiography, which completely resolved shortly afterwards with medical management.

## Discussion

Spinal epidural hematoma results from the rupture of delicate epidural venous plexus veins that are contained between the dural sac and the periosteum of the spinal canal. Because the dural sac and periosteum are adherent in the ventral aspect of the canal, most epidural bleeds occur in the dorsal aspect where the epidural space is relatively slack with loose fatty tissue [12]. Spinal epidural hematomas cause acute compression of the spinal cord, which can present with pain, motor weakness, sensory loss, and bowel and/or bladder dysfunction. The symptoms usually develop within hours to days of the inciting event [13]. MR imaging is the gold standard for diagnosis, evaluation of extension, and assessment of the severity of cord compression [14].

Chiropractic spinal manipulation involves high velocity thrusting forces applied to the spinal column [15-16]. It is hypothesized that these sudden low amplitude thrusts can generate spikes of intraspinal pressure sufficient to cause the rupture of the delicate dural veins [17]. Due to the close temporal association of CSMT and acute onset of severe neck pain and neurologic symptoms, it is likely that our patient's epidural hemorrhage was a result of the chiropractic treatment he underwent the day prior to his presentation. Domenicucci, et al. demonstrated increased signal intensity in T1 and T2 weighted images as well as gadolinium enhancement of the paravertebral muscle mass consistent with a hemorrhagic contusion. They concluded that this finding was suggestive of excessive force attributable to improper CSMT [1]. In our case, the patient’s MR imaging did not demonstrate evidence of excessive force. This suggests that even if performed properly, standard CSMT can generate enough intraspinal force to precipitate rupture of a spinal AVM that is already under pressure within the canal. We, therefore, caution that treatment with CSMT should be avoided in patients who have a known spinal AVM causing neck pain and myeloradiculopathy prior to surgical/endovascular obliteration.      

Although the spontaneous resolution of a cervical SEH has been reported in the literature [18-19], the consensus is that symptomatic SEH is a surgical emergency and early operation is the key to neurological recovery. McQuarrie observed that surgical treatment provided within the first 36 hours of symptom onset results in a favorable prognosis of adequate recovery. Beyond that time period, chances of a good outcome fall drastically, and if surgery is delayed over 3.5 days, patients fail to recover any voluntary movement by five months [20]. Based on the experience of two cases, Song and Lee reported poor outcomes if surgery was performed after 24 hours from symptom onset [9]. 

Lawton, et al. investigated a series of 30 patients who suffered from a spinal epidural hematoma and examined the relationship between surgical timing and neurological outcome. The average postoperative Frankel grade decreased from 4.7 to 3.7 in patients operated on in less than six hours versus after 24 hours, and there was a corresponding decrease in rate of complete recovery from 67% to 12%. Better outcomes were seen in patients operated on within 12 hours of symptom onset [21].

## Conclusions

Spinal epidural hematoma is a rare surgical emergency that is associated with trauma, spondylosis, coagulopathy, and iatrogenic procedures, such as spine surgery and lumbar punctures. Presentation following manipulation of the spine is even more rare. To the best of our knowledge, SEH following CSMT in a patient with an undiagnosed cervical spinal AVM has never been reported. It is possible that even properly performed, non-excessive CSMT may precipitate spinal AVM rupture; therefore, patients with known spinal AVM should avoid CSMT for symptom relief prior to surgical/endovascular intervention. Regardless of etiology, SEH causes severe compression of the cord, which can result in irreversible neurological deficits. Because the likelihood of complete neurological recovery is inversely correlated with the time to surgical intervention, a high index of suspicion, early recognition, and evacuation is essential.
